# An efficient method to remove mixed Gaussian and random-valued impulse noise

**DOI:** 10.1371/journal.pone.0264793

**Published:** 2022-03-03

**Authors:** Mengdi Xing, Guorong Gao

**Affiliations:** College of Sciences, Northwest A&F University, Yangling, P. R. China; University of Bradford, UNITED KINGDOM

## Abstract

Mixed Gaussian and Random-valued impulse noise (RVIN) removal is still a big challenge in the field of image denoising. Existing denoising algorithms have defects in denoising performance and computational complexity. Based on the improved “detecting then filtering” strategy and the idea of inpainting, this paper proposes an efficient method to remove mixed Gaussian and RVIN. The proposed algorithm contains two phases: noise classification and noise removal. The noise classifier is based on Adaptive center-weighted median filter (ACWMF), three-sigma rule and extreme value processing. Different from the traditional “detecting then filtering” strategy, a preliminary RVIN removal step is added to the noise removal phase, which leads to three steps in this phase: preliminary RVIN removal, Gaussian noise removal and final RVIN removal. Firstly, RVIN is processed to obtain a noisy image approximately corrupted by Gaussian noise only. Subsequently, Gaussian noise is re-estimated and then denoised by Block Matching and 3D filtering method (BM3D). At last, the idea of inpainting is introduced to further remove RVIN. Extensive experimental results demonstrate that the proposed method outperforms quantitatively and visually to the state-of-the-art mixed Gaussian and RVIN removal methods. In addition, it greatly shortens the computation time.

## 1. Introduction

Noise is everywhere in life, not only in the signal will have noise interference [1, 2], noise removal in the image is also a big challenge. Noise is an inevitable random phenomenon in images, which may degrades the visual quality of images, distorts its original content and burdens any preprocessing step that may be undertaken [3]. Meanwhile, the existence of the noise will also bring adverse effects to image analysis, recognition and interpretation in the later stage. In practice, the most common types of noise introduced into images can be considered as Gaussian noise and impulse noise [4]. Impulse noise can be further divided into salt and pepper noise (SPN) and random-valued impulse noise (RVIN), where RVIN is more general than SPN because SPN can be converted to RVIN in some cases. There are many studies on the single noise image denoising of Gaussian noise or RVIN, such as the methods in [5–9] for Gaussian noise removal and the algorithms in [10–14] for RVIN reduction. However, in most cases, the noise contained in natural images may not be single, it can be regarded as a mixture of Gaussian noise and impulse noise. Based on the generality of RVIN, this paper aims to study the noise removal of mixed Gaussian and RVIN.

Due to the totally different distributions of Gaussian noise and RVIN, applying the single noise removal methods directly to remove mixed noise always leads to undesirable results. Some important denoising methods for mixed Gaussian and RVIN noise removal based on “detecting then filtering” strategy [4] has been found in recent years [15–22]. “Detecting then filtering” is a strategy that detecting the impulse noise firstly, then abandoning them and using the remaining pixels to estimate the original noise-free image. In 2005, Roman Garnett et al. [15] proposed a trilateral filter (TF) based on this strategy to remove Gaussian and impulse noise simultaneously. They defined a statistic named rank-ordered absolute differences (ROAD) to detect impulse noise and further incorporated it into the bilateral filter (BF) for mixed removal. TF can remove mixed noise effectively. But when the noise level is a bit high, the denoised image is undesirable due to the lack of useful information in a local filtering neighbor. Inspired by the “detecting then filtering” strategy, a two-phase approach [16] for mixed Gaussian and impulse noise removal was proposed in 2008. First, it identifies the pixels that are likely to be corrupted by the impulse noise, and removes them from the dataset. Then, the image is deblurred and denoised simultaneously using essentially the noise-free data. However, the denoised image performs not very well because there are still some Gaussian noises in the image. In 2011, Li Bing et al. [17] put forward a new method which called non-local mixed noise filter (NLMNF) for mixed noise removal. Though it can remove Gaussian noise and RVIN simultaneously, it suffers from unstable results when the images are corrupted with a high level of mixed noise. In 2012, a method named ROR-NLM [18] was also presented based on “detecting then filtering” strategy. By using the statistic robust outlyingness ratio (ROR) to detect noise, the non-local means (NLM) filter [19] is extended to remove mixed noise. However, due to the use of an iterative framework, this algorithm will take a lot of time. Zhou et al. [20] proposed their method of restoring the image corrupted by mixed Gaussian and RVIN in 2013. It used a detector based on Bayesian theory to detect noises and then trained an adaptive overcomplete dictionary to get the final recovered image. However, this algorithm is also time consuming due to the training of the structured dictionary. In 2016, a new method named customized block-wise non-local means filter (CBNLMF) [21] was proposed. This filter is based on block-wise NLM. Unfortunately, this algorithm does not perform well for images with a lot of texture details. Later, Zhou et al. [21] proposed an image denoising algorithm combining CBNLMF and sparse representation (SR) technique to remove mixed Gaussian and RVIN. It used a CBNLMF-based detector to classify mixed noises and then removed them by SR. Nevertheless, this algorithm still needs some time to operate. Yamaguchi, T et al. [22] proposed a new mixed noise removal method in 2017, which utilizing Direction Weighted Median filter (DWMF) [23] and Block Matching and 3D filtering method (BM3D) [5]. This method leads to high performance of mixed noise removal. However, the denoising effect still needs improve, especially at high noise levels.

Due to the limitations of the methods mentioned above [15–22], it is essential to design an efficient method to deal with the mixed Gaussian and RVIN noise removal problem. Hence, this paper proposes a new algorithm based on improved “detecting then filtering” strategy and the idea of inpainting. It effectively addresses the mixed Gaussian and RVIN noise removal problem and greatly reduces the computation time. In general, the proposed denoising algorithm is divided into two phases: (i) noise classification phase and (ii) noise removal phase. In the noise classification phase, we put forward a novel mixed noise classifier which based on adaptive center weighted median filter (ACWMF) [24] and three-sigma rule [25]. In addition, the extreme values similar to impulse noise are also processed. Compared with classifiers in directional weighted median filter (DWMF) [23], 2013 Zhou’s [20] and 2016 Zhou’s [21], ours is fast and efficient. In the noise removal phase, different from the traditional “detecting then filtering” strategy, we add a preliminary RVIN removal step to this phase, which leads to three steps: (i) preliminary RVIN removal step, (ii) Gaussian noise removal step and (iii) final RVIN removal step. At the first step, the adaptive median filter (AMF) is used on those noisy pixels corrupted by RVIN for initial processing to obtain a noisy image approximately corrupted by Gaussian noise only. At the second step, the Gaussian noise level is re-estimated first and then the Gaussian noises, which are in the approximate Gaussian noise image obtained at the previous step, are removed by employing BM3D [5] algorithm. At the last step, the RVIN is removed for the second time based on the idea of inpainting. Extensive experimental results show that our proposed algorithm not only work well in mixed Gaussian and RVIN noise removal, but also has minimal computation time compared with NLMNF [17], CBNLMF [21], 2013 Zhou’s [20], 2016 Zhou’s [21] and 2017 Yam’s [22] methods.

The rest of the paper is organized as follows. Section 2 introduces the noise model of mixed Gaussian and RVIN. In Section 3, the proposed novel noise classification scheme for classifying mixed noise pixels is described. Then, the proposed noise removal scheme based on the improved “detecting then filtering” strategy and the idea of inpainting is presented in Section 4. The summary of the whole proposed denoising algorithm is given in Section 5. In Section 6, experimental results and discussions are shown in the noise classification phase and noise removal phase, respectively. Finally, the conclusion is drawn in Section 7.

## 2. Mixed Gaussian and RVIN noise model

The noise removal of a natural image corrupted by mixed Gaussian and RVIN remains a challenging problem in the field of image denoising. In general, images are destroyed first by Gaussian noise in the acquisition process, and then by impulse noise in the transmission process [3]. Therefore, the noise model of mixed Gaussian and RVIN, which denoted as *Y*, is expressed as follows:

$$Y\left(i,j\right)=\left\{ \begin{array}{cc} R(i,j) & ,p=p_{0}\\ X(i,j)+n(i,j) & ,p=1-p_{0} \end{array}\right.$$ (1)

where (*i*, *j*) (*i* = 1, …, *M*. *j* = 1, …, *N*.) denotes the location of a pixel in the whole image, *Y*(*i*, *j*) refers to the gray value of noisy image pixel, *X*(*i*, *j*) is the gray value of original noise-free image, *R*(*i*, *j*) shows gray value of uniformly distributed noise in range [*X*_min_, *X*_max_] while *p*_0_ is the probability of the RVIN, and *n*(*i*, *j*) stands for the noisy value which is drawn from a Gaussian distribution with zero mean and *σ* standard deviation.

In this paper, we use *σ*&*p*_0_ to represent the mixed noise level, where *σ* denotes the standard deviation of Gaussian noise and *p*_0_ refers to the probability of the RVIN. With the mixed noise model, the goal of our image denoising method is to restore the unknown true pixels from the pixels in *Y* which are corrupted by mixed Gaussian and RVIN. Since the distribution of mixed noise cannot be described by a fixed function, different noise pixels should be considered differently. Therefore, we first propose a noise classification algorithm to separate mixed noise pixels.

## 3. Proposed noise classification scheme

The purpose of the noise classification phase is to separate the additive Gaussian noise and the RVIN in the image, so as to further process the two kinds of noise separately. In our noise classification scheme, a good initial denoised image is obtained on the basis of ACWMF [24] firstly. Then, according to the feature of mixed noise, three-sigma rule is employed to get the classification result. Finally, the extreme pixel values of similar impulses are processed to achieve better classification. The proposed noise classifier is efficient and accurate. In Section 3.1, the acquisition of initial denoised image ${\hat{X}}^{\left(ACWMF\right)}$ based on ACWMF is described. The proposed noise classification algorithm is generalized in Section 3.2.

### 3.1. Acquisition of initial denoised image ${\hat{X}}^{\left(ACWMF\right)}$ based on ACWMF

The first step of our proposed noise classification scheme is to acquire the initial denoised image ${\hat{X}}^{\left(ACWMF\right)}$, where the superscript “ACWMF” stands for the adaptive center weighted median filter. Here, the ACWMF algorithm proposed in [24] is chosen to generate ${\hat{X}}^{\left(ACWMF\right)}$ from *Y*. Motivated by the two advantages of ACWMF’s strong noise removal capability and less time consumption, it can ensure the high efficiency of our mixed noise classifier.

ACWMF devises a novel adaptive operator, which forms estimates based on the differences between the current pixels and the outputs of center-weighted median filter (CWMF) [24] with varied center weights. The details are as follows.

Consider a window *W* defined symmetrically around the image coordinates of the current pixel, which is described as:

W={(m,n)|−a≤m≤a,−b≤n≤b} (2)

where *a*, *b* are positive integer and the window size is defined as 2*L* + 1 (*L* > 0). Throughout the following discussion, unless otherwise stated, the window size is assumed to be 3 × 3 (i.e. *a* = *b* = 1, 2*L* + 1 = 9).

Let *X*(*i*, *j*) be the gray value of original noise-free image pixel at position (*i*, *j*). The gray value of mixed noise image pixel *Y*(*i*, *j*) is obtained from the mixed noise model. The output of CWMF [26], in which a weight adjustment is applied to the target noise image pixel *Y*(*i*, *j*) at position (*i*, *j*) within the sliding window in order to obtain the denoised image, can be described as:

$${\hat{X}}^{\omega }(i,j)=median(Y^{\omega }(i,j))$$ (3)

where

$$Y^{\omega }(i,j)=\left\{Y(i-m,j-n),\omega \;◊\;Y(i,j)|(m,n)\in W,(m,n)\neq (0,0)\;\right\}$$ (4)

In the above equations, *Y*(*i*, *j*) is the gray value of current pixel (*i*, *j*) in mixed noise image, ${\hat{X}}^{\omega }(i,j)$ refers to the output of CWMF which represents the estimated gray value of original noiseless image *X*(*i*, *j*), *ω* = 2*k* + 1 denotes the center weight and *k* is the nonnegative integer. When *ω* = 1, the standard median filter which expressed as ${\hat{X}}^{1}(i,j)$ is obtained. Besides, operator ◊ represents the repetition operation and *W* is the window defined above.

Define *d*_*k*_ as the differences between the output of CWMF and noise pixels, the differences *d*_*k*_ is given by:

$$d_{k}=\left|{\hat{X}}^{\omega }(i,j)-Y(i,j)\right|=\left|{\hat{X}}^{2k+1}(i,j)-Y(i,j)\right|$$ (5)

where *k* = 0, 1, …, *L*−1. It is easy to know that *d*_*k*_ ≤ *d*_*k*−1_ (*k* ≥ 1) based upon the derivation shown in [27]. These differences provide information about the likelihood of corruption for the current pixel.

Consider four thresholds *T*_*k*_ (*k* = 0,1,2,3), which are described as:

$$T_{k}=s\cdot MAD+\delta_{k}$$ (6)

where *T*_*k*_ < *T*_*k*−1_ (*k* ≥ 1), and MAD is a robust estimate of dispersion [28], which is given by:

$$MAD=median\left\{\left|Y(i-m,j-n)-{\hat{X}}^{1}(i,j)\right||(m,n)\in W\right\}$$ (7)

where ${\hat{X}}^{1}(i,j)$ is the output of standard median filter.

In this paper, parameters are set to [*δ*_0_, *δ*_1_, *δ*_2_, *δ*_3_] = [40, 25, 10, 5] and *s* = 0.6, which are selected following the suggestions in original literature [24]. From the simulation conducted on a broad variety of images, it has been observed that the selection above yields satisfactory results.

In a word, the ACWMF can be realized as follows:

$${\hat{X}}^{\left(ACWMF\right)}(i,j)=\left\{ \begin{array}{l} {\hat{X}}^{1}(i,j),if\exists k,d_{k}>T_{k}\\ Y(i,j),otherwise \end{array}\right.$$ (8)

where ${\hat{X}}^{\left(ACWMF\right)}(i,j)$ denotes the output of ACWMF, *T*_*k*_ refers to a set of thresholds (*k* = 0, 1, …, *L* − 1) where *T*_*k*_ < *T*_*k*−1_ (*k* ≥ 1), and *d*_*k*_ is the difference defined above.

Specifically, if *d*_*k*_ < *T*_*k*_, the pixels are regarded as noise and then treated with SMF. Otherwise, the current pixel remains unchanged. The algorithm of ACWMF is summarized in **Algorithm 1**.

**Algorithm 1. The algorithm of ACWMF**.

**Input**: a mixed noise image *Y* corrupted by mixed Gaussian and RVIN

**Output**: a denoised image ${\hat{X}}^{\left(ACWMF\right)}$

 **Step1**: Calculate *d*_*k*_ value according to **Formula** (5);

 **Step2**: Calculate corresponding thresholds *T*_*k*_ according to **Formulas** (6) and (7);

 **Step3**: For every pixel, we estimate its true gray value ${\hat{X}}^{\left(ACWMF\right)}(i,j)$ based on **Formula** (8). After processing all pixels, the denoised image ${\hat{X}}^{\left(ACWMF\right)}$ is obtained.

### 3.2. Proposed mixed noise classifier

Based on the output of ACWMF, an absolute difference image between the noisy image *Y* and denoised image ${\hat{X}}^{\left(ACWMF\right)}$ is obtained:

$$I_{d}=abs(Y-{\hat{X}}^{\left(ACWMF\right)})$$ (9)

Here *abs*(·) represents the operation of getting the absolute value for every element in a matrix.

Since ${\hat{X}}^{\left(ACWMF\right)}$ is an initial estimation of the original noise-free image *X*, there are two types of values in *I*_*d*_: One is the absolute value of Gaussian noise and the other is the difference between impulse noise and the noise-free values. Three-sigma rule [25] is a classical statistical method through which a simple threshold can use to differentiate the noisy pixels. Let *l* be a label matrix corresponding to *Y*. Then,

$$l(i,j)=\left\{ \begin{array}{l} 1,if\;I_{d}(i,j)\leq 3\sigma \\ 0,if\;I_{d}(i,j)>3\sigma \end{array}\right.$$ (10)

where “1” represents the pixel corrupted by Gaussian noise, and “0” indicates that the pixel is distorted by RVIN.

For better classification, two kinds of extreme values are processed: the maximum and the minimum values of the mixed noise image, which can be recognized as impulse noise. It can be realized as follows:

$$l(i,j)=0,\;if\;Y=Y_{\text{max}}\;\text{or}\;Y_{\text{min}}$$ (11)

where *Y*_max_ and *Y*_min_ represent the maximum and minimum value in the mixed noise image *Y*, respectively. The proposed novel mixed noise classifier is concluded in **Algorithm 2**.

**Algorithm 2. The proposed mixed noise classifier**.

**Input**: a mixed noise image *Y* corrupted by mixed Gaussian and RVIN

**Output**: a label matrix *l* labeling the type of every pixel in *Y*

 **Step1**: Operate ACWMF filter for *Y* as **Algorithm 1**. and an initial denoised image ${\hat{X}}^{\left(ACWMF\right)}$ is generated;

 **Step2**: Construct an absolute difference image *I*_*d*_ by $I_{d}=abs(Y-{\hat{X}}^{\left(ACWMF\right)})$;

 **Step3**: Generate a label matrix *l* according to **Formulas** (10) and (11).

## 4. Proposed noise removal scheme

After the noise classification phase, we separate the Gaussian noise and RVIN effectively. Then, a noise removal scheme is needed to remove the mixed noise to restore the image. To improve the “denoising then filtering” strategy, we added an initial step which named preliminary RVIN removal in the denoising stage. Therefore, the noise removal phase comprises three steps: (i) preliminary RVIN removal, (ii) Gaussian noise removal, and (iii) final RVIN removal.

### 4.1. Preliminary RVIN removal

Since the RVIN only destroys part of the pixels in the image while Gaussian noise destroys all the pixels, it is inevitably to use the Gaussian noise in the surrounding neighborhood to estimate the original pixel when removing the RVIN. So that there is still an error in the estimation. Based on the above analysis, the first step that we perform in the noise removal phase is the preliminary RVIN removal. Since the impulse noise destroys the pixel information, the purpose of the preliminary RVIN removal step is to provide a pixel value containing image information to the position of the impulse, which facilitates the Gaussian noise removal in the next step. In the case of mixed Gaussian and RVIN, it is important to first suppress the RVIN [29], so an impulse removal filter which is robust to Gaussian noise is need to use. Therefore, the method applied in the preliminary RVIN removal step needs to meet the following two conditions:

1. The algorithm is robust to the existence of Gaussian noise;
2. The algorithm is simple with fast speed.

**Fig 1:**
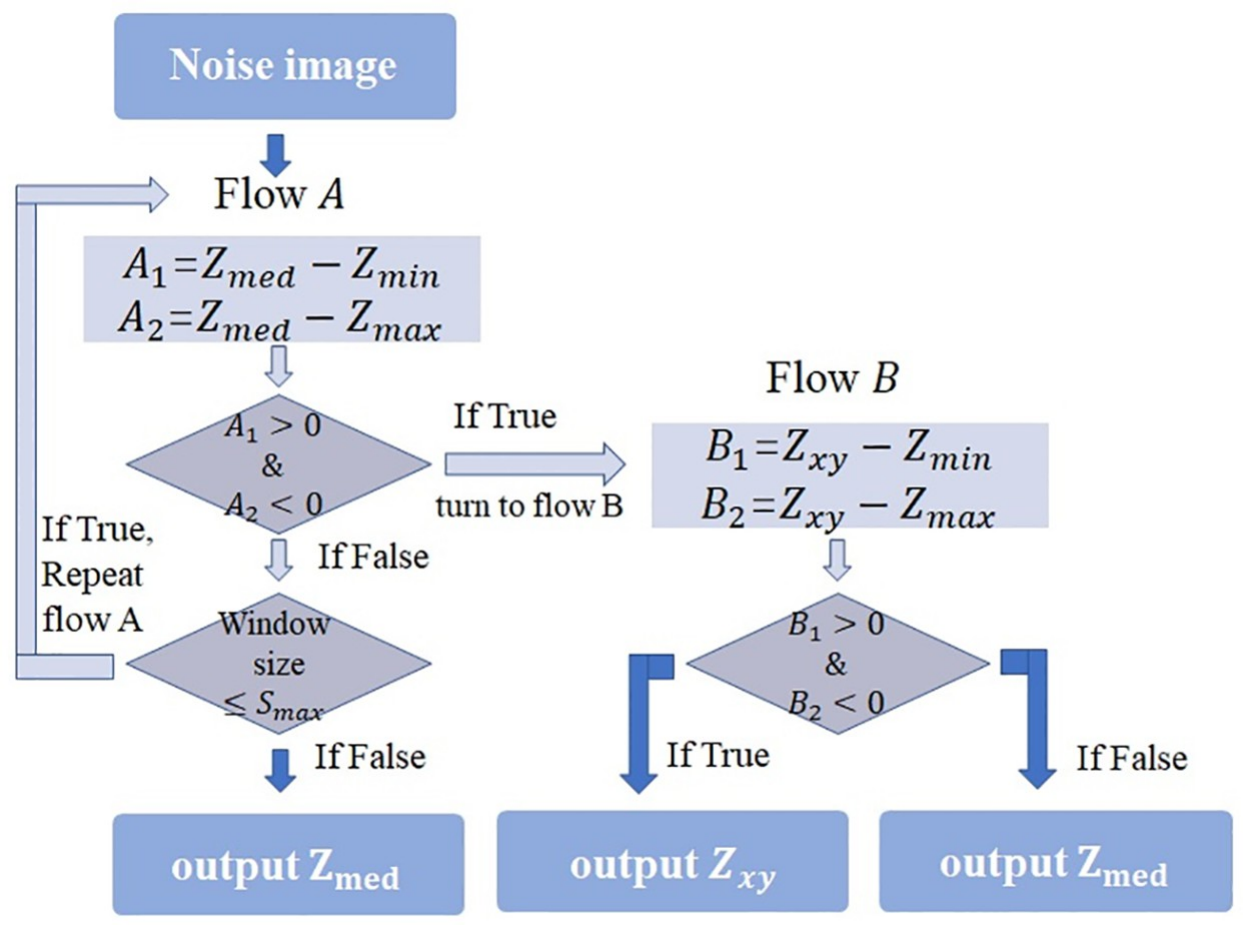
The flowchart of AMF algorithm.

Based on the above two conditions, the adaptive median filter (AMF) algorithm mentioned in Fig 1 is chosen for preliminary RVIN removal. At this time, the pixel value at the RVIN position is estimated by using pixels in its neighborhoods, which can be approximately regarded as Gaussian noise, and then it can be removed as Gaussian noise in the following steps.

AMF algorithm works with flow *A* and flow *B*, the flowchart is in Fig 1:

where *W*(*i*, *j*) is a window centered at (*i*, *j*) with (2*a* + 1)×(2*b* + 1) as window size. *Z*_*med*_ is the median of the grayscale value in the window *W*(*i*, *j*). In the same way, we define *Z*_max_ as the maximum and *Z*_min_ as the minimum, *Z*(*i*, *j*) represents the grayscale value at coordinate (*i*, *j*), *W*_max_ denotes the maximum allowed window in *W*(*i*, *j*). In this paper, we apply initial window as *W*_3×3_ (i.e. *a* = *b* = 1) and *W*_max_ as *W*_5×5_ (i.e. *a* = *b* = 2) which are selected based on a broad variety of images simulation. Then, the output of AMF is named as ${\hat{X}}^{\left(AMF\right)}$.

In a word, the proposed preliminary RVIN removal steps can be realized as follows:

$${\hat{X}}^{\left(pre\right)}(i,j)=\left\{ \begin{array}{cc} {\hat{X}}^{\left(AMF\right)}(i,j) & ,if\;pixel(i,j)\;is\;recoginzed\;as\;RVIN\\ Y(i,j) & ,if\;pixel(i,j)\;is\;recoginzed\;as\;Gaussian \end{array}\right.$$ (12)

The pixels identified as RVIN in the noise classification phase are processed by AMF algorithm in Fig 1, while the pixels classified into Gaussian noise remain unchanged. Through this step, we obtain a noisy image approximately corrupted only by Gaussian noise through preliminary processing of RVIN, which named as approximate Gaussian noisy image.

### 4.2. Gaussian noise removal

As the second step of noise removal phase, Gaussian noise removal is also very important. Due to the fact that Gaussian noise destroys all pixels in the image, it is a great challenge to completely remove Gaussian noise. The proposed Gaussian noise removal step is further divided into two small steps: (i) Gaussian noise level re-estimation and (ii) Gaussian noise removal by BM3D.

#### 4.2.1. Gaussian noise level re-estimation

Since the approximate Gaussian noisy image is obtained through the preliminary RVIN removal step, in which the RVIN after preliminary processing is approximately regarded as Gaussian noise, it is necessary to re-estimate the Gaussian noise level.

Here, the Gaussian noise level estimation method proposed in [30] is applied to re-estimate the Gaussian noise level of the approximate Gaussian noisy image obtained in the preliminary RVIN removal step. The method is described as follows.

When re-estimating Gaussian noise level, it is essential to re-estimate standard deviation *σ* of the Gaussian noise. So first divide the whole approximate Gaussian noisy image into four sub-images(sub-groups), and the sample variances of these four sub-groups are calculated respectively. Then, the estimation of Gaussian noise variance ${\hat{\sigma }}^{2}$ is re-estimated by averaging all the sample variances:

$${\hat{\sigma }}^{2}=\frac{1}{{\left(M\times N\right)}^{2}}\sum_{i=1}^{M}\sum_{j=1}^{N}s{\left(i,j\right)}^{2}$$ (13)

where *M*×*N* refers to the size of approximate Gaussian noisy image and *s*(*i*, *j*)^2^ represents the sample variances of sub-groups. At last, the estimation of the standard deviation $\hat{\sigma }$ we need is obtained by taking the square root of the ${\hat{\sigma }}^{2}$. For more detailed information about this Gaussian noise level estimation method, please refer to the literature [30].

#### 4.2.2. Gaussian noise removal by BM3D

**Fig 2:**
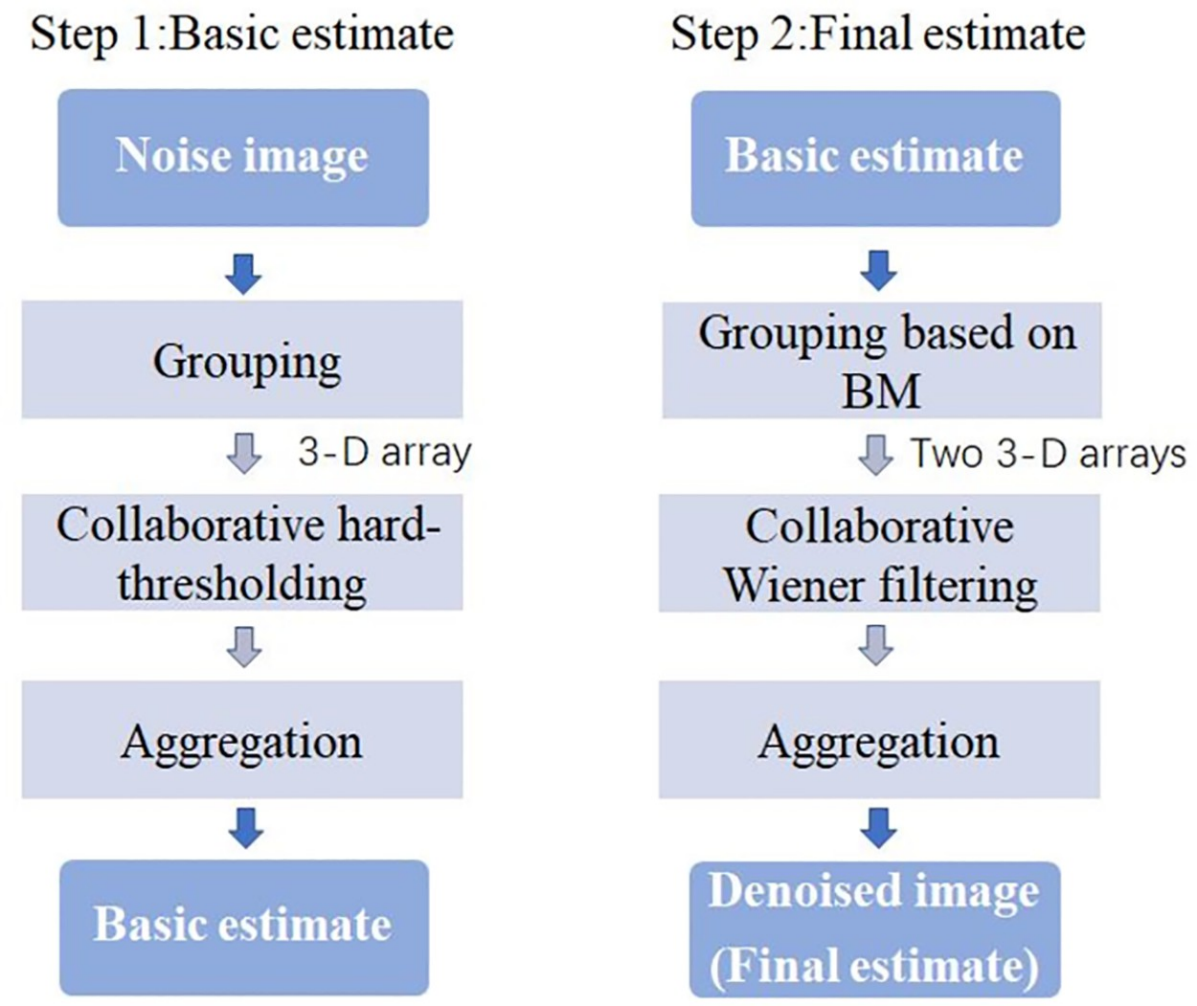
The flowchart of BM3D algorithm.

After re-estimating the Gaussian noise level, the BM3D algorithm proposed in [5] is used to remove Gaussian noise in this step. BM3D algorithm is a filtering algorithm based on 3-D transform and block-wise estimation. It consists of two steps: (i) basic estimate and (ii) final estimate. In the first step, the noisy image to be processed is divided into fixed size sub-groups and each block of image is estimated one by one. Blocks are grouped according to how similar they are to the currently processed one and then, they are stacked together in a 3-D array(group). Then, the collaborative hard-thresholding is performed to the formed 3D group and finally the basic estimate of the true original image from the overlapping blocks is computed by aggregation. The second step is using the basic estimate, performing improved grouping and collaborative wiener filter. Above all, use the basic estimate obtained in the first step to estimate each block for the second time. Through blocking matching (BM), the locations of the blocks similarly to the currently processed one can be found. After BM, two 3-D arrays (groups) are obtained: one is from the noisy image and the other is from the basic estimate. Then, perform collaborative Wiener filter on the two 3-D arrays mentioned above. At last, the final estimate is computed by the weighted average of the overlapping block estimations which obtained by aggregation. More algorithm details are presented in S1 File. The output of the BM3D algorithm is named as ${\hat{X}}^{\left(BM3D\right)}$ and the flowchart of this algorithm is shown in Fig 2:

### 4.3. Final RVIN removal

The third step of the noise removal phase is the final RVIN noise removal step. It is assumed that all the Gaussian noises in the image have been removed after the Gaussian noise removal step. Obviously, the location of the RVIN is already known in the noise classification phase. Hence, the final RVIN noise removal step only deals with the pixels at the RVIN position, while the pixels in the neighbors used for estimating the original pixel are all regarded as clean pixels. Pursuing the denoising performance, the idea of image inpainting is introduced into this step to remove RVIN. The main idea of image inpainting is to use the observed information to reconstruct the damaged or obscured area [31]. In this step, by cleverly combining the idea of inpainting, the processing of damaged areas is transformed into the processing of the points of RVIN. The inpainting method based on mixed median [32] is chosen to modify and perform here. The details are in **Algorithm 3**.

**Algorithm 3. The image inpainting algorithm based on the mixed median**.

**Input**: ${\hat{X}}^{\left(BM3D\right)}$ & label matrix *l*

**Output**: ${\hat{X}}^{\left(inpainting\right)}$

 **Step1**: Initialize the set of RVIN pixels $Y^{\left(RVIN\right)}=(1-l)\times {\hat{X}}^{\left(BM3D\right)}$. For every RVIN pixel in *Y*^(*RVIN*)^, if there is at least one noise-free pixel in *W*_3×3_ (the window of 3×3 size), eliminate RVIN pixels from *W*_3×3_ and compute the median of the remaining pixels, then update *Y*^(*RVIN*)^. Else if do this process in *W*_7×7_ window as the same.

 **Step2**: Initialize a dynamic window whose size starts from 3×3 and increases by *d* = 1. For every RVIN pixel in *Y*^(*RVIN*)^ obtained from the step1, when there is at least one noise-free pixel in the considered dynamic window, compute the maximum repetitive pixels values, and evaluate the median of it, then update *Y*^(*RVIN*)^. End this step until *d* > *d*_max_, where *d*_max_ = (min{*M*, *N*} − 1)/2. Finally, the ${\hat{X}}^{\left(inpainting\right)}$ is obtained.

In a word, the proposed final RVIN removal step can be realized as follows:

$${\hat{X}}^{\left(final\right)}(i,j)=\left\{ \begin{array}{cc} {\hat{X}}^{\left(inpainting\right)}(i,j) & ,if\;pixel(i,j)\;is\;recoginzed\;as\;RVIN\\ {\hat{X}}^{(BM3D)\;}(i,j) & ,\;pixel(i,j)\;remain \end{array}\right.$$ (14)

The pixels identified as RVIN in the noise classification phase are processed by inpainting method in **Algorithm 3**, while the remaining pixels remain unchanged at the last step. Through this step, the final denoised image ${\hat{X}}^{\left(final\right)}$ is obtained.

## 5. Summary of the proposed denoising scheme

**Fig 3:**
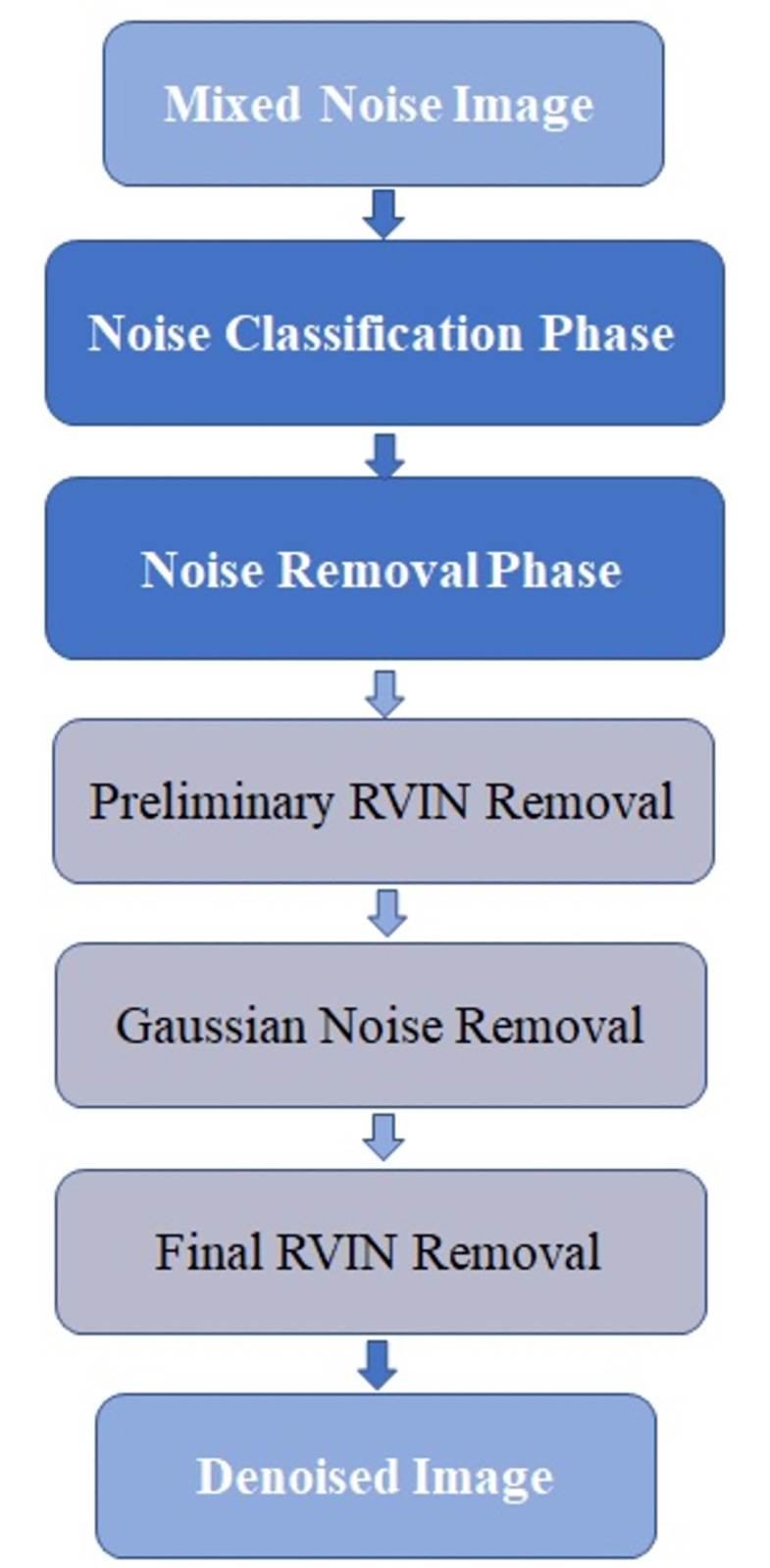
The proposed algorithm flowchart.

In general, the proposed denoising algorithm is divided into two phases: (i) noise classification phase and (ii) noise removal phase. The proposed novel mixed noise classifier is based on ACWMF, three-sigma rules and extreme value processing. Based on the improved “detecting then filtering” strategy, the noise removal phase contains three steps: (i) preliminary RVIN removal step, (ii) Gaussian noise removal step, and (iii) final RVIN removal step. The idea of inpainting is introduced to the final RVIN removal step. The flowchart of the whole proposed denoising algorithm is in Fig 3.

## 6. Experimental results and discussion

To assess the capability of the proposed algorithm for mixed Gaussian and RVIN noise removal, comprehensive numerical results and visual quality are compared with other state-of-the-art methods. Now, the proposed algorithm is tested from two perspectives. One is the performance of noise classification in Section 6.1, the other is the image denoising performance in Section 6.2.

### 6.1. Comparison of noise classification

For good performance, the capability of noise classification is exceedingly important. The performance of the proposed noise classifier is compared with three state-of-the-art ones coping with mixed Gaussian and RVIN. The compared methods are DWMF [23], 2013 Zhou’s [20] and 2016 Zhou’s [21].

In this section, four noise classification evaluation indexes are introduced. They are Miss_error [21], False_error [21], Total_error [21] and Computation time. Miss_error is the number of pixels distorted by RVIN but wrongly classified into Gaussian noise. False_error counts the number of pixels corrupted by Gaussian noise but wrongly identified as RVIN. Total_error equals to Miss_error plus False_error. Computation time refers to the running time of the algorithm. By considering these four evaluation indexes in a comprehensive way, if Total_error and computation time of the proposed classification algorithm are the minimum among these comparison methods mentioned above, we have the confidence to employ it into our whole denoising algorithm.

**Fig 4:**
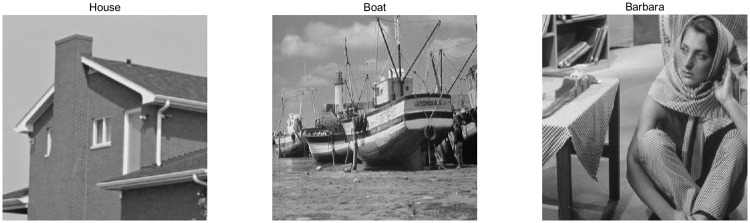
Test images used in this paper.

To test our classifier in the noise classification phase, three standard test images: “House”, “Boat” and “Barbara” are chosen. The selected images are shown in Fig 4, they are 512 × 512 size which have a dynamic range of pixels varying from 0 to 255. It is reasonable to select these three images as the test images for they contain different level of edges and textures.

In Tables 1–3, the results of the noise classification evaluation indexes for noisy “House”, “Boat” and “Barbara” images are presented. These noisy images are corrupted with different mixed noise levels *σ* = 15, 20, 25, 30 & *p*_0_ = 0.2, 0.3. The simulated additive Gaussian noise is with zero mean and four standard deviations *σ* = 15, 20, 25, 30. Meanwhile, the noise densities *p*_0_ of RVIN are 0.2 and 0.3. The bold numbers in the tables are the smallest ones in the related columns.

**Table 1:** Comparison of classifiers for noisy “House” with σ = 15,20,25,30&p_0_ = 0.2,0.3.

**Classifier**	**σ = 15&p**_**0**_ **= 0.2**				**σ = 15&p**_**0**_ **= 0.3**			
	**Miss_error**	**False_error**	**Total_error**	**Time/s**	**Miss_error**	**False_error**	**Total_error**	**Time/s**
DWMF	51850	3001	54851	3.30	76254	9529	85783	3.35
2013zhou’s	**1088**	2370	3458	121.06	2745	4734	7479	106.07
2016zhou’s	1941	**532**	2473	31.95	6795	**830**	7625	31.90
ours	1147	858	**2005**	**0.009**	**1928**	1049	**2977**	**0.008**
**Classifier**	**σ = 20&p**_**0**_ **= 0.2**				**σ = 20&p**_**0**_ **= 0.3**			
	**Miss_error**	**False_error**	**Total_error**	**Time/s**	**Miss_error**	**False_error**	**Total_error**	**Time/s**
DWMF	51783	3046	54829	3.36	76177	9599	85776	4.06
2013zhou’s	**2044**	2639	4683	143.58	**4727**	5821	10548	106.18
2016zhou’s	6571	**523**	7094	32.38	14104	**680**	14784	74.13
ours	3624	716	**4340**	**0.009**	5812	788	**6600**	**0.009**
**Classifier**	**σ = 25&p**_**0**_ **= 0.2**				**σ = 25&p**_**0**_ **= 0.3**			
	**Miss_error**	**False_error**	**Total_error**	**Time/s**	**Miss_error**	**False_error**	**Total_error**	**Time/s**
DWMF	51642	3188	54830	3.32	75964	9771	85735	3.29
2013zhou’s	**6423**	3254	**9677**	134.73	**10358**	6557	**16915**	137.20
2016zhou’s	13606	**518**	14124	94.84	24540	**558**	25098	92.34
ours	12190	609	12799	**0.008**	18487	583	19070	**0.009**
**Classifier**	**σ = 30&p**_**0**_ **= 0.2**				**σ = 30&p**_**0**_ **= 0.3**			
	**Miss_error**	**False_error**	**Total_error**	**Time/s**	**Miss_error**	**False_error**	**Total_error**	**Time/s**
DWMF	51459	3373	54832	3.38	75639	10083	85722	3.38
2013zhou’s	**12651**	2885	15536	115.55	**18971**	6749	25720	103.84
2016zhou’s	15602	**474**	16076	96.67	28771	587	29358	99.51
ours	14402	500	**14902**	**0.008**	21935	**533**	**22468**	**0.009**

**Table 2:** Comparison of classifiers for noisy “Boat” with σ = 15,20,25,30&p_0_ = 0.2,0.3.

**Classifier**	**σ = 15&p**_**0**_ **= 0.2**				**σ = 15&p**_**0**_ **= 0.3**			
	**Miss_error**	**False_error**	**Total_error**	**Time/s**	**Miss_error**	**False_error**	**Total_error**	**Time/s**
DWMF	51267	3750	55017	3.24	75454	10459	85913	3.26
2013zhou’s	3196	5908	9104	101.28	6047	9623	15670	101.10
2016zhou’s	4477	**1803**	**6280**	32.69	9958	**2325**	12283	31.91
ours	**3041**	3921	6962	**0.0090**	**4561**	3918	**8479**	**0.0082**
**Classifier**	**σ = 20&p**_**0**_ **= 0.2**				**σ = 20&p**_**0**_ **= 0.3**			
	**Miss_error**	**False_error**	**Total_error**	**Time/s**	**Miss_error**	**False_error**	**Total_error**	**Time/s**
DWMF	51025	4591	55616	3.37	75047	11187	86234	3.35
2013zhou’s	**4392**	4944	9336	101.69	8190	8432	16622	141.77
2016zhou’s	6240	1301	7541	58.95	12229	**1625**	13854	79.66
ours	4385	**2395**	**6780**	**0.0086**	**6675**	2571	**9246**	**0.0079**
**Classifier**	**σ = 25&p**_**0**_ **= 0.2**				**σ = 25&p**_**0**_ **= 0.3**			
	**Miss_error**	**False_error**	**Total_error**	**Time/s**	**Miss_error**	**False_error**	**Total_error**	**Time/s**
DWMF	50690	5432	56122	3.26	74544	12081	86625	3.26
2013zhou’s	**5425**	4651	10076	102.08	10153	8636	18789	113.54
2016zhou’s	8221	**1104**	9325	93.70	18506	**1198**	19704	106.79
ours	6022	1714	**7736**	**0.0083**	**9279**	1844	**11123**	**0.0086**
**Classifier**	**σ = 30&p**_**0**_ **= 0.2**				**σ = 30&p**_**0**_ **= 0.3**			
	**Miss_error**	**False_error**	**Total_error**	**Time/s**	**Miss_error**	**False_error**	**Total_error**	**Time/s**
DWMF	50388	6343	56731	3.36	74044	12935	86979	3.37
2013zhou’s	**6880**	4621	11501	188.68	**12711**	9337	22048	101.62
2016zhou’s	12317	**852**	13169	126.15	26359	**945**	27304	96.52
ours	9749	1333	**11082**	**0.0080**	15072	1474	**16546**	**0.0077**

**Table 3:** Comparison of classifiers for noisy “Barbara” with σ = 15,20,25,30&p_0_ = 0.2,0.3.

**Classifier**	**σ = 15&p**_**0**_ **= 0.2**				**σ = 15&p**_**0**_ **= 0.3**			
	**Miss_error**	**False_error**	**Total_error**	**Time/s**	**Miss_error**	**False_error**	**Total_error**	**Time/s**
DWMF	51663	3190	54853	3.31	75974	9801	85775	3.29
2013zhou’s	**4150**	6925	11075	164.53	8128	12716	20844	103.20
2016zhou’s	7378	**2073**	**9451**	34.07	16121	**3250**	19371	40.52
ours	4340	9915	14255	**0.0078**	**6702**	9107	**15809**	**0.0085**
**Classifier**	**σ = 20&p**_**0**_ **= 0.2**				**σ = 20&p**_**0**_ **= 0.3**			
	**Miss_error**	**False_error**	**Total_error**	**Time/s**	**Miss_error**	**False_error**	**Total_error**	**Time/s**
DWMF	51342	3808	55150	3.30	75445	10544	85989	3.38
2013zhou’s	**7278**	5228	12506	181.63	12978	11072	24050	173.99
2016zhou’s	10857	**1505**	**12362**	33.04	20034	**2246**	22280	77.79
ours	7364	5472	12836	**0.0086**	**11352**	5232	**16584**	**0.0082**
**Classifier**	**σ = 25&p**_**0**_ **= 0.2**				**σ = 25&p**_**0**_ **= 0.3**			
	**Miss_error**	**False_error**	**Total_error**	**Time/s**	**Miss_error**	**False_error**	**Total_error**	**Time/s**
DWMF	50828	4890	55718	3.39	74615	11842	86457	3.40
2013zhou’s	**9448**	5277	14725	192.73	16630	9960	26590	173.54
2016zhou’s	13590	**1447**	15037	92.30	25839	**1828**	27667	93.34
ours	10180	3361	**13541**	**0.0077**	**15515**	3078	**18593**	**0.0084**
**Classifier**	**σ = 30&p**_**0**_ **= 0.2**				**σ = 30&p**_**0**_ **= 0.3**			
	**Miss_error**	**False_error**	**Total_error**	**Time/s**	**Miss_error**	**False_error**	**Total_error**	**Time/s**
DWMF	50294	6149	56443	3.37	73852	13218	87070	3.39
2013zhou’s	**11655**	4847	16502	103.17	**20217**	10013	30230	103.40
2016zhou’s	17632	**1120**	18752	115.33	32313	**1432**	33745	93.42
ours	14167	2127	**16294**	**0.0087**	21564	2087	**23651**	**0.0121**

From the data in Tables 1–3, it is obvious that our proposed novel mixed noise classifier can guarantee the lowest classification errors which defined as Total_error and the shortest computation time at almost all the noise level, even when the noise level is as high as *σ* = 30 & *p*_0_ = 0.3. So, there is no doubt that the proposed noise classifier is efficient and accurate. The good noise classification performance benefits from the good initial denoised image obtained by ACWMF, classical statistical principles of three-sigma rules and the treatment of extreme values. Hence, we have the confidence to employ the proposed noise classification scheme into our whole denoising algorithm.

### 6.2. Comparison of noise removal

#### 6.2.1. Image quality metrics introduction

For quantitative performance evaluation, two image quality metrics are employed. They are: peak signal to noise ratio (PSNR) and structural similarity index (SSIM).

PSNR is one of the most classical metrics for image quality assessment (IQA). It is computed as the ratio of the peak intensity value of the reference image to the RMS reconstruction error relative to the reference. Also, its values are usually given in logarithmic decibel units(dB). PSNR provides a global statistical similarity over intensity distribution [9]. PSNR is used as quantitative measurement to compare the proposed method with different well-known techniques. The PSNR is defined as:

$$PSNR=10{\text{log}}_{10}\left(\frac{255}{\sqrt{MSE}}\right)$$ (15)

where MSE is mean squared error and is defined as:

$$MSE=\frac{1}{M\times N}\sum_{i=1}^{M}\sum_{j=1}^{N}{\left(X(i,j)-{\hat{X}}^{\left(final\right)}(i,j)\right)}^{2}$$ (16)

In the above equations, *X* is the original noise-free image while ${\hat{X}}^{\left(final\right)}$ is the output denoised image, and *M* × *N* is the size of the image. The larger the PSNR value is, the better the denoising effect is.

SSIM [33] is based on the computation of three terms, which named as the luminance term, the contrast term and the structural term. The overall index is a multiplicative combination of the three terms. The SSIM is defined as:

$$SSIM(x,y)={\left[l(x,y)\right]}^{\alpha }{\left[c(x,y)\right]}^{\beta }{\left[s(x,y)\right]}^{\gamma }$$ (17)

$$l(x,y)=\frac{2\mu_{x}\mu_{y}+C_{1}}{\mu_{x}^{2}+\mu_{y}^{2}+C_{1}}$$ (18)

$$c(x,y)=\frac{2\sigma_{x}\sigma_{y}+C_{2}}{\sigma_{x}^{2}+\sigma_{y}^{2}+C_{2}}$$ (19)

$$s(x,y)=\frac{\sigma_{xy}+C_{3}}{\sigma_{x}\sigma_{y}+C_{3}}$$ (20)

where *μ*_*x*_, *μ*_*y*_, *σ*_*x*_, *σ*_*y*_ and *σ*_*xy*_ are the local means, standard deviations, and cross-covariance for images *x*, *y*. If *α* = *β* = *γ* = 1, and *C*_3_ = *C*_2_ / 2, the SSIM simplifies to:

$$SSIM(x,y)=\frac{\left(2\mu_{x}\mu_{y}+C_{1})(2\sigma_{xy}+C_{2}\right)}{\left(\mu_{x}^{2}+\mu_{y}^{2}+C_{1})(\sigma_{x}^{2}+\sigma_{y}^{2}+C_{2}\right)}$$ (21)

Substitute the images *x*, *y* with the original clean image *X* and the output denoised image ${\hat{X}}^{\left(final\right)}$ when calculating SSIM. Similarly, the higher the value of SSIM is, the better the denoising effect of the algorithm is.

#### 6.2.2. Numerical results and visual quality

The denoising performance of the proposed method is tested on three test images mentioned above in Fig 4. Consistent with the noise classification phase, we add different mixed noise level *σ* = 15, 20, 25, 30 & *p*_*0*_ = 0.2, 0.3 to the above three test images in order to obtain mixed noise images. The performance of our whole denoising algorithm is shown on numerical results and visual quality respectively. To validate the superiority of the proposed method, its performance is compared in terms of PSNR, SSIM, computation time and visual quality of the denoised images using the various methods available in literature such as NLMNF [17], CBNLMF [21], 2013 Zhou’s [20], 2016 Zhou’s [21] and 2017 Yam’s [22]. The parameters in these algorithms are set to values suggested by the authors.

All algorithms have been implemented using MATLAB 2019a (The MathWorks, Inc., Natick, MA, USA). Also, data processing was carried out on a Dell workstation system with a 2.80 GHz Intel Core i7 processor and 16 GB memory.

PSNR and SSIM comparisons of the proposed method and other baseline algorithms on test images corrupted by mixed Gaussian and RVIN with different noise levels are tabulated in Tables 4–6, respectively.

**Table 4:** Comparison of PSNR and SSIM for noisy “House” with σ = 15,20,25,30&p_0_ = 0.2,0.3.

**Algorithm**	**σ = 15&p**_**0**_ **= 0.2**		**σ = 15&p**_**0**_ **= 0.3**		**σ = 20&p**_**0**_ **= 0.2**		**σ = 20&p**_**0**_ **= 0.3**	
	**PSNR**	**SSIM**	**PSNR**	**SSIM**	**PSNR**	**SSIM**	**PSNR**	**SSIM**
NLMNF	32.19	0.851	26.10	0.722	30.99	0.816	25.14	0.667
CBNLMF	31.75	0.845	25.07	0.669	29.83	0.777	25.55	0.681
2013zhou’s	40.41	0.882	35.42	0.829	39.09	0.858	34.31	0.787
2016zhou’s	38.66	0.885	30.95	0.714	35.37	0.786	30.11	0.619
2017Yam’s	28.21	0.639	27.44	0.747	29.72	0.774	27.85	0.782
ours	**42.75**	**0.925**	**41.09**	**0.903**	**40.55**	**0.891**	**38.39**	**0.845**
**Algorithm**	**σ = 25&p**_**0**_ **= 0.2**		**σ = 25&p**_**0**_ **= 0.3**		**σ = 30&p**_**0**_ **= 0.2**		**σ = 30&p**_**0**_ **= 0.3**	
	**PSNR**	**SSIM**	**PSNR**	**SSIM**	**PSNR**	**SSIM**	**PSNR**	**SSIM**
NLMNF	29.56	0.768	24.14	0.603	28.09	0.714	22.91	0.535
CBNLMF	28.85	0.767	23.13	0.572	27.38	0.702	22.00	0.499
2013zhou’s	36.31	0.792	32.80	0.715	34.49	0.746	31.01	0.645
2016zhou’s	32.93	0.686	27.69	0.477	32.35	0.712	26.85	0.468
2017Yam’s	30.43	0.796	28.15	0.779	31.01	0.796	28.56	0.775
ours	**38.05**	**0.858**	**35.42**	**0.798**	**36.95**	**0.850**	**34.25**	**0.789**

**Table 5:** Comparison of PSNR and SSIM for noisy “Boat” with σ = 15,20,25,30&p_0_ = 0.2,0.3.

**Algorithm**	**σ = 15&p**_**0**_ **= 0.2**		**σ = 15&p**_**0**_ **= 0.3**		**σ = 20&p**_**0**_ **= 0.2**		**σ = 20&p**_**0**_ **= 0.3**	
	**PSNR**	**SSIM**	**PSNR**	**SSIM**	**PSNR**	**SSIM**	**PSNR**	**SSIM**
NLMNF	26.87	0.712	23.09	0.601	26.22	0.685	22.53	0.566
CBNLMF	26.94	0.737	22.68	0.603	26.01	0.696	22.78	0.582
2013zhou’s	34.17	0.767	31.09	0.685	33.43	0.739	30.58	0.660
2016zhou’s	33.51	0.769	28.68	0.638	32.45	0.728	28.54	0.619
2017Yam’s	26.91	0.497	26.06	0.531	27.64	0.550	26.35	0.540
ours	**35.86**	**0.811**	**34.96**	**0.794**	**34.90**	**0.786**	**33.89**	**0.763**
**Algorithm**	**σ = 25&p**_**0**_ **= 0.2**		**σ = 25&p**_**0**_ **= 0.3**		**σ = 30&p**_**0**_ **= 0.2**		**σ = 30&p**_**0**_ **= 0.3**	
	**PSNR**	**SSIM**	**PSNR**	**SSIM**	**PSNR**	**SSIM**	**PSNR**	**SSIM**
NLMNF	25.52	0.654	21.86	0.527	24.78	0.619	21.16	0.486
CBNLMF	25.13	0.656	21.27	0.512	24.37	0.618	20.54	0.466
2013zhou’s	32.79	0.715	29.96	0.631	32.02	0.685	29.36	0.603
2016zhou’s	31.21	0.676	26.70	0.517	29.98	0.620	25.57	0.445
2017Yam’s	27.98	0.551	26.55	0.534	28.22	0.551	26.82	0.531
ours	**34.00**	**0.758**	**32.84**	**0.728**	**32.90**	**0.726**	**31.40**	**0.680**

**Table 6:** Comparison of PSNR and SSIM for noisy “Barbara” with σ = 15,20,25,30&p_0_ = 0.2,0.3.

**Algorithm**	**σ = 15&p**_**0**_ **= 0.2**		**σ = 15&p**_**0**_ **= 0.3**		**σ = 20&p**_**0**_ **= 0.2**		**σ = 20&p**_**0**_ **= 0.3**	
	**PSNR**	**SSIM**	**PSNR**	**SSIM**	**PSNR**	**SSIM**	**PSNR**	**SSIM**
NLMNF	25.90	0.780	21.51	0.645	25.10	0.743	21.00	0.601
CBNLMF	25.36	0.788	20.94	0.635	24.35	0.735	21.16	0.624
2013zhou’s	34.03	0.840	29.84	0.735	33.09	0.803	29.12	0.693
2016zhou’s	31.68	0.805	26.80	0.641	30.52	0.759	26.80	0.627
2017Yam’s	26.02	0.490	25.29	0.525	26.69	0.552	25.53	0.544
ours	**34.51**	**0.866**	**33.20**	**0.837**	**34.02**	**0.847**	**32.51**	**0.810**
**Algorithm**	**σ = 25&p**_**0**_ **= 0.2**		**σ = 25&p**_**0**_ **= 0.3**		**σ = 30&p**_**0**_ **= 0.2**		**σ = 30&p**_**0**_ **= 0.3**	
	**PSNR**	**SSIM**	**PSNR**	**SSIM**	**PSNR**	**SSIM**	**PSNR**	**SSIM**
NLMNF	24.32	0.700	20.48	0.557	23.54	0.656	19.86	0.511
CBNLMF	23.77	0.696	20.12	0.550	23.05	0.650	19.49	0.503
2013zhou’s	32.17	0.770	28.48	0.657	31.23	0.734	27.89	0.616
2016zhou’s	29.83	0.717	25.69	0.546	28.83	0.662	24.90	0.495
2017Yam’s	26.99	0.560	25.73	0.650	27.21	0.559	25.95	0.542
ours	**33.30**	**0.822**	**31.61**	**0.775**	**32.28**	**0.784**	**30.36**	**0.727**

The bold numbers in Tables 4–6 are the best values in the related columns. It is clear from the tables that the proposed denoising algorithm always generate the highest PSNR and SSIM. So, it can demonstrate that the proposed method can generate the best denoised results whatever noisy image is and whatever noise level is. Especially in “Barbara” with the most detailed textures, NLMNF, CBNLMF, 2013 Zhou’s, 2016 Zhou’s and 2017 Yam’s do not perform well, but ours still get best results in the values of PSNR and SSIM.

Now, we discuss the time complexity of our whole mixed noise algorithm. As shown in Tables 7–9, five other related algorithms are chosen for comparison. Here, we operate the MATLAB codes of the above six algorithms on the same platform- MATLAB 2019a. The computer is equipped with Dell workstation system with a 2.80 GHz Intel Core i7 processor and 16 GB memory. The data in Tables 7–9 is the CPU consuming time of each algorithm. The bold numbers are the smallest time in the related columns. Obviously, with the help of improved “detecting then filtering” strategy and the idea of inpainting, the proposed method consumes the least time among the six algorithms. In other words, our algorithm obtains the best denoising performance in shortest time.

**Table 7:** Comparison of Time (seconds) for noisy “House” with σ = 15,20,25,30&p_0_ = 0.2,0.3.

Algorithm	σ = 15&p_0_ = 0.2	σ = 15&p_0_ = 0.3	σ = 20&p_0_ = 0.2	σ = 20&p_0_ = 0.3	σ = 25&p_0_ = 0.2	σ = 25&p_0_ = 0.3	σ = 30&p_0_ = 0.2	σ = 30&p_0_ = 0.3
NLMNF	114.9	126.0	101.8	103.8	113.8	107.1	106.7	104.4
CBNLMF	32.1	31.7	32.5	87.1	93.7	93.8	114.1	131.5
2013zhou’s	348.6	263.1	261.1	272.3	289.8	393.2	310.8	286.5
2016zhou’s	53.27	123.2	64.9	155.2	127.7	280.9	135.1	442.9
2017Yam’s	11.0	11.2	10.8	11.2	11.0	11.1	11.0	10.9
ours	**2.50**	**2.45**	**3.49**	**2.44**	**2.64**	**2.62**	**3.14**	**2.68**

**Table 8:** Comparison of Time (seconds) for noisy “Boat” with σ = 15,20,25,30&p_0_ = 0.2,0.3.

Algorithm	σ = 15&p_0_ = 0.2	σ = 15&p_0_ = 0.3	σ = 20&p_0_ = 0.2	σ = 20&p_0_ = 0.3	σ = 25&p_0_ = 0.2	σ = 25&p_0_ = 0.3	σ = 30&p_0_ = 0.2	σ = 30&p_0_ = 0.3
NLMNF	100.2	112.4	101.8	102.8	103.0	101.4	206.8	103.3
CBNLMF	43.6	37.6	32.2	91.0	91.2	104.4	93.4	95.7
2013zhou’s	274.8	285.2	342.1	335.1	256.7	256.4	257.8	271.7
2016zhou’s	104.7	383.2	178.9	373.5	173.8	530.8	267.2	754.3
2017Yam’s	10.7	10.7	10.9	10.8	11.0	10.8	11.0	10.7
ours	**2.30**	**3.20**	**2.42**	**2.39**	**2.38**	**2.40**	**3.17**	**3.19**

**Table 9:** Comparison of Time (seconds) for noisy “Barbara” with σ = 15,20,25,30&p_0_ = 0.2,0.3.

Algorithm	σ = 15&p_0_ = 0.2	σ = 15&p_0_ = 0.3	σ = 20&p_0_ = 0.2	σ = 20&p_0_ = 0.3	σ = 25&p_0_ = 0.2	σ = 25&p_0_ = 0.3	σ = 30&p_0_ = 0.2	σ = 30&p_0_ = 0.3
NLMNF	100.5	126.2	101.1	114.1	99.9	103.3	101.6	102.0
CBNLMF	46.0	48.3	32.5	74.7	96.9	142.0	93.2	145.6
2013zhou’s	459.4	466.5	419.3	341.9	374.3	345.3	387.0	290.9
2016zhou’s	294.9	660.7	484.1	710.5	312.6	738.0	349.7	874.7
2017Yam’s	11.4	11.0	10.9	10.9	10.9	10.9	10.7	11.0
ours	**2.26**	**3.06**	**2.32**	**2.31**	**2.29**	**3.21**	**2.41**	**2.27**

**Fig 5:**
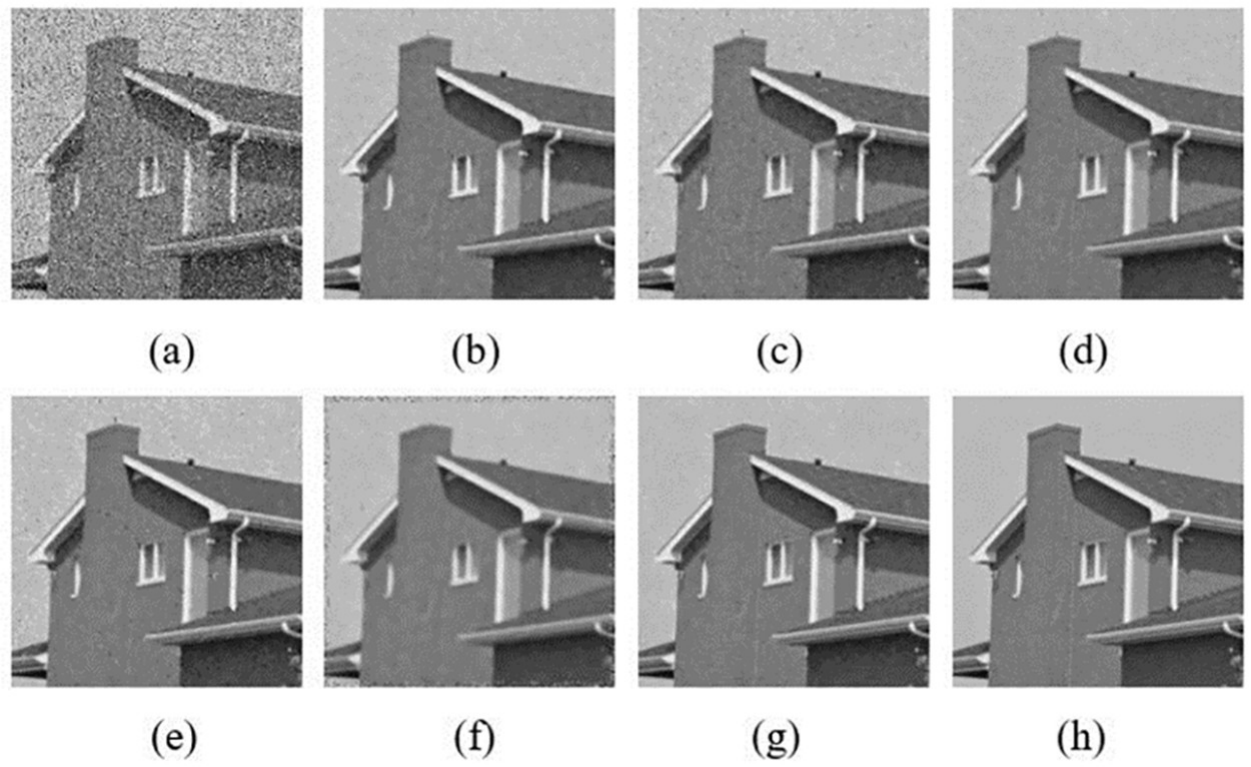
Results of different algorithm in denoised “House” with σ = 20&p_0_ = 0.2: (a) Mixed noise image; (b) NLMNF; (c) CBNLMF; (d) 2013 Zhou’s; (e) 2016 Zhou’s; (f) 2017 Yam’s; (g) Proposed; (h) Original image.

**Fig 6:**
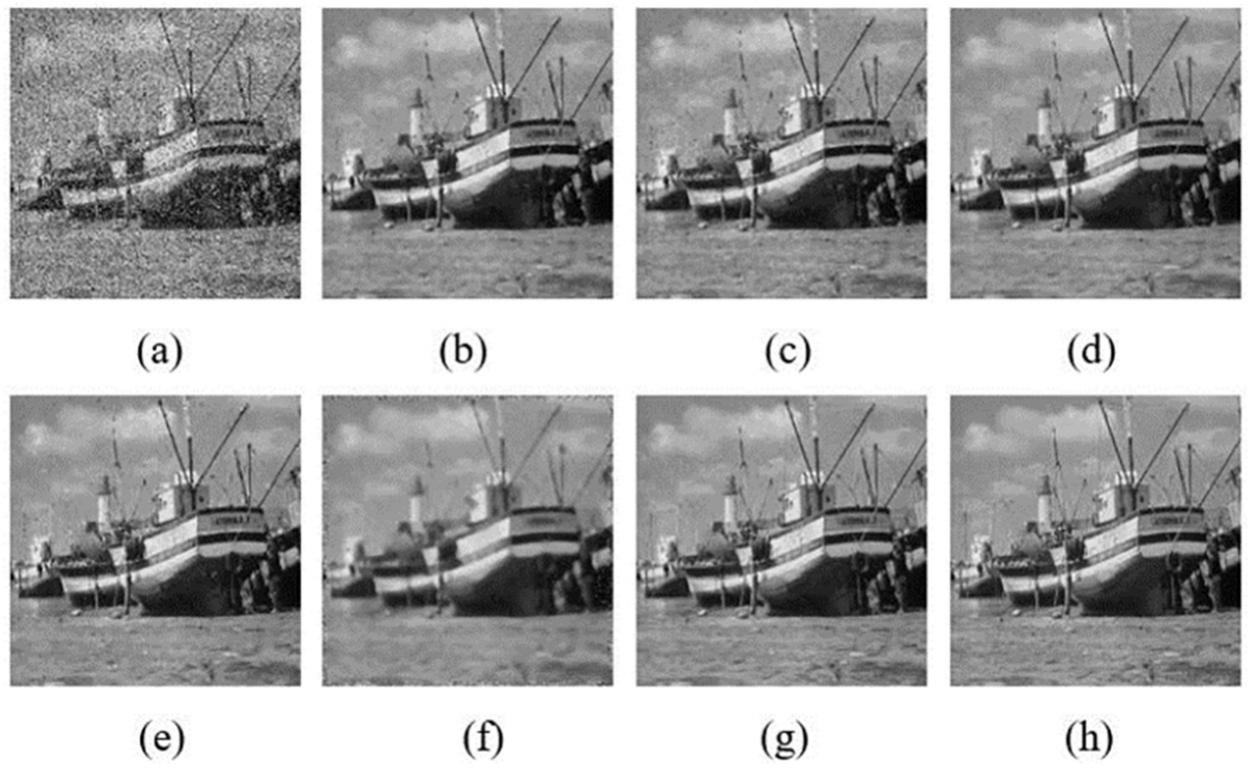
Results of different algorithm in denoised “Boat” with σ = 20&p_0_ = 0.2: (a) Mixed noise image; (b) NLMNF; (c) CBNLMF; (d) 2013 Zhou’s; (e) 2016 Zhou’s; (f) 2017 Yam’s; (g) Proposed; (h) Original image.

**Fig 7:**
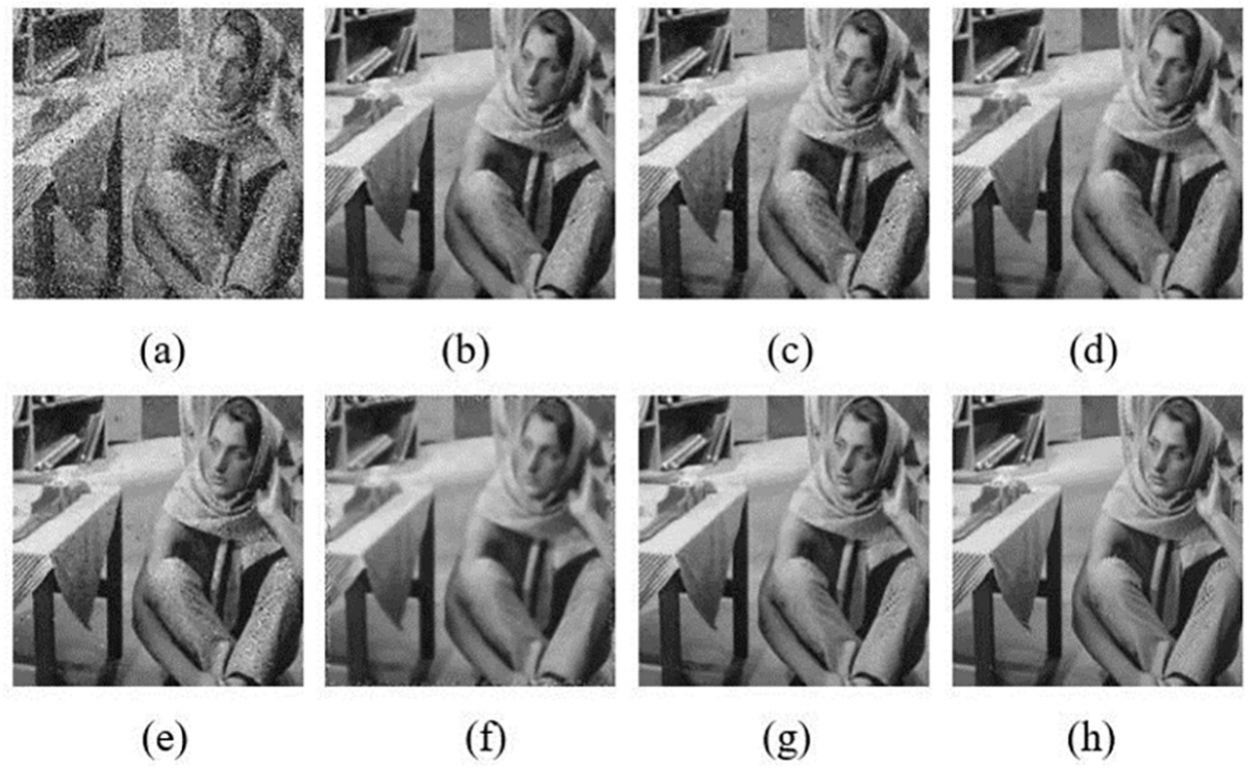
Results of different algorithm in denoised “Barbara” with σ = 20&p_0_ = 0.2: (a) Mixed noise image; (b) NLMNF; (c) CBNLMF; (d) 2013 Zhou’s; (e) 2016 Zhou’s; (f) 2017 Yam’s; (g) Proposed; (h) Original image.

In order to have a close-up observation to the noise removal images, some typical results are chosen to show. They are denoised “House”, “Boat” and “Barbara” with the mixed noise level of *σ* = 20 & *p*_*0*_ = 0.2. The denoised images compared with NLMNF [17], CBNLMF [21], 2013 Zhou’s [20], 2016 Zhou’s [21] and 2017 Yam’s [22] are shown in Figs 5–7.

Fig 5 shows the comparisons on visual results from the test cases on “House”. It can be clearly observed that there are more fine structures remained in the “House” of the proposed denoising algorithm shown in Fig 5e, especially some of the textures on the exterior walls. However, the “House” images denoised by NLMNF, CBNLMF, 2013 Zhou’s, 2016 Zhou’s and 2017 Yam’s which shown in Fig 5b–5f respectively do not perform well. The denoised images obtained by CBNLMF and 2016 Zhou’s are distorted a lot. 2017 Yam’s owns fast speed but the image after denoising is a bit blurry. Furthermore, there are still some noises in these five “House” images.

Similar conclusions can be drawn from Figs 6 and 7, the texture details and edge structure of the denoised image obtained by the algorithm in this paper are clearer and the visibility of our denoised result is better. For instance, the boat and wave in Fig 6g is more clearer than that in Fig 6b–6f. Especially in Fig 7, although there are most texture details in “Barbara” image, the proposed algorithm in Fig 7g still preserves these textures well.

## 7. Conclusion

In this paper, we propose an efficient method for mixed Gaussian and RVIN noise removal. Experimental results verify that the proposed algorithm performs well not only in noise classification phase but also in noise removal phase compared with several state-of-the-art methods. The proposed mixed noise removal method has achieved good denoising performance both in numerical results and visual quality, and greatly reduced the computation time.

## Supporting information

S1 File The BM3D algorithm in details. (PDF)

S1 Table Abbreviation comparison. (PDF)

## References

[1] Zhang Q, Wang H. A Novel Data-based Stochastic Distribution Control for Non-Gaussian Stochastic Systems. IEEE Transactions on Automatic Control. 2021.

[2] Yin X, Zhang Q., Wang H. and Ding Z. Rbfnn-based minimum entropy filtering for a class of stochastic nonlinear systems. IEEE Transactions on Automatic Control. 2019;65(1):pp.376–381.

[3] Mafi M, Martin H, Cabrerizo M, Andrian J, Barreto A, Adjouadi M. A comprehensive survey on impulse and Gaussian denoising filters for digital images. Signal Processing. 2019;157:236–260.

[4] Chen LL, Licheng L, Philip Chen, C. L. A robust bi-sparsity model with non-local regularization for mixed noise reduction. Information Sciences. 2016;354:101–111.

[5] Dabov K, Foi A, Katkovnik V, Egiazarian K. Image denoising by sparse 3-D transform-domain collaborative filtering. IEEE Trans Image Process. 2007;16(8):2080–2095. doi: 10.1109/tip.2007.901238 17688213

[6] Shao L, Yan R, Li X, Liu Y. From heuristic optimization to dictionary learning: a review and comprehensive comparison of image denoising algorithms. IEEE Trans Cybern. 2014;44(7):1001–1013. doi: 10.1109/TCYB.2013.2278548 24002014

[7] Guo Q, Zhang C, Zhang Y, Liu H. An Efficient SVD-Based Method for Image Denoising. IEEE Transactions on Circuits and Systems for Video Technology. 2016;26(5):868–880.

[8] Rakhshanfar M, Amer MA. Efficient cascading of multi-domain image Gaussian noise filters. Journal of Real-Time Image Processing. 2019;17(5):1183–1195.

[9] Wu G, Luo S, Yang Z. Optimal weighted bilateral filter with dual-range kernel for Gaussian noise removal. IET Image Processing. 2020;14(9):1840–1850.

[10] Wu TCaHR. Space variant median filters for the restoration of impulse noise corrupted images. IEEE Transactions on Circuits and Systems for Video Technology. 2001:784–789.

[11] Schulte S, De Witte V, Nachtegael M, Van der Weken D, Kerre EE. Fuzzy random impulse noise reduction method. Fuzzy Sets and Systems. 2007;158(3):270–283.

[12] Turkmen I. A new method to remove random-valued impulse noise in images. AEU—International Journal of Electronics and Communications. 2013;67(9):771–779.

[13] Gupta V, Chaurasia V, Shandilya M. Random-valued impulse noise removal using adaptive dual threshold median filter. Journal of Visual Communication and Image Representation. 2015;26:296–304.

[14] Nadeem M, Hussain A, Munir A, Habib M, Naseem MT. Removal of random valued impulse noise from grayscale images using quadrant based spatially adaptive fuzzy filter. Signal Processing. 2020;169.

[15] Garnett R., Huegerich T., Chui C., He W. A universal noise removal algorithm with an impulse detector. IEEE TRANSACTIONS ON IMAGE PROCESSING. 2005;14(11):1747–1754. doi: 10.1109/tip.2005.857261 16279175

[16] Cai JF, Chan RH, Nikolova M. Two-phase approach for deblurring images corrupted by impulse plus Gaussian noise. Inverse Probl Imaging. 2008;2(2):187–204.

[17] Li B, QuanSheng L, Xu JW, Luo XJ. A new method for removing mixed noises. Science China Information Sciences. 2011;54(1):51–59.

[18] Xiong B, Yin Z. A universal denoising framework with a new impulse detector and nonlocal means. IEEE Trans Image Process. 2012;21(4):1663–1675. doi: 10.1109/TIP.2011.2172804 22020688

[19] Buades A, Bartomeu C, Morel J.M. A Review of Image Denoising Algorithms, with a New One. Multiscale Modeling & Simulation. 2005;4(2):490–530.

[20] Zhou Y, Ye Z, Xiao Y. A restoration algorithm for images contaminated by mixed Gaussian plus random-valued impulse noise. Journal of Visual Communication and Image Representation. 2013;24(3):283–294.

[21] Yingyue Zhou M L, Su Xu, Hongbin Zang, Hongsen He, Qiang Li, Jin Guo. An image denoising algorithm for mixed noise combining nonlocal means filter and sparse representation technique. Journal of Visual Communication and Image Representation. 2016;41:74–86.

[22] Yamaguchi T, Suzuki A, Ikehara M. Detail Preserving Mixed Noise Removal by DWM Filter and BM3D. IEICE Trans Fundam Electron Commun Comput Sci. 2017;E100A(11):2451–2457.

[23] Dong Y, Xu S. A New Directional Weighted Median Filter for Removal of Random-Valued Impulse Noise. IEEE Signal Processing Letters. 2007;14(3):193–196.

[24] Wu TCaHR. Adaptive impulse detection using center-weighted median filters. IEEE SIGNAL PROCESSING LETTERS. 2001;8.

[25] Maronna R M R., and Yohar V. Robust Statistics: Theory and Methods. Chichester, UK:Wiley. 2006.

[26] Lee Y.-H., Ko S.-J. Center weighted median filters and their applications to image enhancement. IEEE TransCircuits Syst. 1991;38:984–993.

[27] Chen T., Ma K.-K., and Chen L.-H. Tri-state median filter for image denoising. IEEE TransImage Processing. 1999;8:1834–1838. doi: 10.1109/83.806630 18267461

[28] Hampel F.R., Ronchetti E.M., Rousseeuw P.J., and Stahel W.A. Robust Statistics: The Approach Based on Influence Functions. New York:Wiley. 1986.

[29] Astola SMAKOEDZGJT. Combining the discrete wavelet transforms and rank-order based filters for image restoration. Optical Engineering. 1998.

[30] Li M, Ghosal S. Bayesian Multiscale Smoothing of Gaussian Noised Images. Bayesian Analysis. 2014;9(3):733–758.

[31] Wan W, Liu J. Nonlocal patches based Gaussian mixture model for image inpainting. Applied Mathematical Modelling. 2020;87:317–331.

[32] D. N. H. Thanh, V. B. S. Prasath, L. M. Hieu and H. Kawanaka, “Image Inpainting Method Based on Mixed Median,” 2019 Joint 8th International Conference on Informatics, Electronics & Vision (ICIEV) and 2019 3rd International Conference on Imaging, Vision & Pattern Recognition (icIVPR), 2019, pp. 24–29.

[33] Zhou W, Bovik A. C., Sheikh H. R., and Simoncelli E. P. Image Qualifty Assessment: From Error Visibility to Structural Similarity. IEEE Transactions on Image Processing. 2004;13 (4):600–612. doi: 10.1109/tip.2003.819861 15376593

