# Supplementary material for: An efficient method to remove mixed Gaussian and random-valued impulse noise
Source: PLoS One. 2022 Mar 3;17(3):e0264793. doi: 10.1371/journal.pone.0264793 (PMC8893653; doi:10.1371/journal.pone.0264793)
Supplement: S1 File — (PDF) [file pone.0264793.s001.pdf]

S1 File. The BM3D algorithm in details.

BM3D algorithm[1] is a filtering algorithm based on 3-D transform and block-wise estimation. It consists of two steps: (i) basic estimate and (ii) final estimate.

(i) **Basic estimate**

In the first step, the noise image to be processed is divided into fixed size sub-groups and each block of image is estimated one by one. Group blocks according to how similar they are to the currently processed one, the purpose of grouping is to find similar blocks for target blocks. A Normalized 2D Linear Transform and hard-thresholding are used to preprocess the block distance. The formula is as follows:

$$d(Z_{xR}, Z_x) = \frac{\left\| \Upsilon'(T_{2D}^{ht}(Z_{xR})) - \Upsilon'(T_{2D}^{ht}(Z_x)) \right\|_2^2}{(N_1^{ht})^2} \qquad (1)$$

Where x is the pixel of image X , Z_{xR} is the corresponding target block and Z_x is a searching block. N_1^{ht} is the size of block in this step. Υ' is the hard-thresholding operation and T_{2D}^{ht} is a normalized 2D linear transformation.

The set of similar blocks S_{xR}^{ht} can be found according to the distance, as shown in Equation (2).

$$S_{xR}^{ht} = \left\{ x \in X : d(Z_{xR}, Z_x) \le \tau_{match}^{ht} \right\} \qquad (2)$$

Where τ_{match}^{ht} is a hyperparameter that determines whether blocks are similar. After obtaining S_{xR}^{ht} , a 3-D array(group) $Z_{S_{xR}^{ht}}$ is formed by stacking the matched noisy blocks $Z_{x \in S_{xR}^{ht}}$ to form the group of size $N_1^{ht} \times N_1^{ht} \times \left| S_{xR}^{ht} \right|$.

Then, a 3-D transform T_{3D}^{ht} is applied to the formed group to reduce the noise of similar blocks, the inverse transformation T_{3D}^{ht-1} is used to obtain the similar blocks after processing which denoted as $\hat{Y}_{S_{xR}^{ht}}^{ht}$:

$$\hat{Y}_{S_{xR}^{ht}}^{ht} = T_{3D}^{ht-1}(\Upsilon(T_{3D}^{ht}(Z_{S_{xR}^{ht}}))) \qquad (3)$$

Where T_{3D}^{ht} is the normalized 3D linear transformation and T_{3D}^{ht-1} is the inverse transformation. Υ is the hard-thresholding operation.

Finally compute the basic estimate of the true original image from the overlapping blocks by aggregation.

$$\hat{y}^{basic}(x)=\frac{\sum_{xR\in X}\sum_{x_m\in S_{xR}^{ht}}\omega_{xR}^{ht}\hat{Y}_{xm}^{ht,xR}(x)}{\sum_{xR\in X}\sum_{x_m\in S_{xR}^{ht}}\omega_{xR}^{ht}\chi_{xm}(x)},\forall x\in X \qquad (4)$$

where

$$\omega_{xR}^{ht}=\begin{cases}\frac{1}{\sigma^2 N_{har}^{xR}}, & if\ N_{har}^{xR}\geq 1\\ 1, & \text{otherwise}\end{cases} \qquad (5)$$

Where ω_{xR}^{ht} is the weight , $\chi_{xm}:X\rightarrow\{0,1\}$ and N_{har}^{xR} is the number of non-zero coefficients after hard-thresholding operation in Formula (3).

(ii) **Final estimate**

The second step is using the basic estimate, performing improved grouping and collaborative wiener filter. Above all, use the basic estimate obtained in the first step to estimate each block for the second time.

$$S_{xR}^{wie}=\left\{x\in X:\frac{\left\|\hat{Y}_{xR}^{basic}-\hat{Y}_{x}^{basic}\right\|_2^2}{(N_1^{wie})^2}<\tau_{match}^{wie}\right\} \qquad (6)$$

The set S_{xR}^{wie} is used in order to form two 3-D arrays (groups): one $Z_{S_{xR}^{wie}}$ is from the noisy image and the other $\hat{Y}_{S_{xR}^{wie}}^{basic}$ is from the basic estimate.

Then, the collaborative Wiener filter is performed on the two 3-D arrays mentioned above.

$$\hat{Y}_{S_{xR}^{wie}}^{wie}=T_{3D}^{wie-1}(W_{S_{xR}^{wie}}T_{3D}^{wie}(Z_{S_{xR}^{wie}})) \qquad (7)$$

Where the Wiener shrinkage coefficient is $W_{S_{xR}^{wie}}$:

$$W_{S_{xR}^{wie}}=\frac{\left|T_{3D}^{wie}(\hat{Y}_{S_{xR}^{wie}}^{basic})\right|^2}{\left|T_{3D}^{wie}(\hat{Y}_{S_{xR}^{wie}}^{basic})\right|^2+\sigma^2} \qquad (8)$$

At last, similarly to the Step (i), the final estimate $\hat{y}^{final}$ is computed as Formula (9) and Formula (10):

$$\hat{y}^{final}(x)=\frac{\sum_{xR\in X}\sum_{x_m\in S_{xR}^{wie}}\omega_{xR}^{wie}\hat{Y}_{xm}^{wie,xR}(x)}{\sum_{xR\in X}\sum_{x_m\in S_{xR}^{wie}}\omega_{xR}^{wie}\chi_{xm}(x)},\forall x\in X \qquad (9)$$

Where the weight is:

$$\omega_{xR}^{wie} = \sigma^{-2} \left\| W_{S_{xR}^{wie}} \right\|_2^{-2} \qquad (10)$$

Reference

[1] Kostadin Dabov, Alessandro Foi, Vladimir Katkovnik, and Karen Egiazarian, Image denoising by sparse 3-D transform-domain collaborative filtering, IEEE Trans Image Process, 16(8) (2007) 2080-2095.
