# Supplementary material for: An efficient method to remove mixed Gaussian and random-valued impulse noise
Source: PLoS One. 2022 Mar 3;17(3):e0264793. doi: 10.1371/journal.pone.0264793 (PMC8893653; doi:10.1371/journal.pone.0264793)
Supplement: S1 Table — (PDF) [file pone.0264793.s002.pdf]

S2 Table. Abbreviation comparison.

Abbreviation	Full name
RVIN	Random-valued impulse noise
ACWMF	Adaptive center-weighted median filter
BM3D	Block Matching and 3D filtering method
SPN	Salt and pepper noise
TF	Trilateral filter
BF	Bilateral filter
NLMNF	Non-local mixed noise filter
ROR-NLM	Robust outlyingness ratio- Non-local means filter
CBNLMF	Customized block-wise non-local means filter
SR	Sparse representation
DWMF	Direction Weighted Median filter
PSNR	Peak signal to noise ratio
SSIM	Structural similarity index
